# Interactive effect of genetic susceptibility with height, body mass index, and hormone replacement therapy on the risk of breast cancer

**DOI:** 10.1186/1472-6874-12-17

**Published:** 2012-06-22

**Authors:** Sophia Harlid, Salma Butt, Malin IL Ivarsson, Jorunn Erla Eyfjörd, Per Lenner, Jonas Manjer, Joakim Dillner, Joyce Carlson

**Affiliations:** 1 Department of Medical Microbiology, Lund University, Malmö, Sweden; 2 Department of Clinical Chemistry, Lund University, Malmö, Sweden; 3 Department of Surgery, Lund University, Malmö, Sweden; 4 Region Skånes Biobank, Wallenberg Laboratory, Malmö, Sweden; 5 Cancer Research Laboratory, Faculty of Medicine, University of Iceland, Reykjavik, Iceland; 6 Department of Oncology, Norrlands University Hospital, Umeå, Sweden; 7 Labmedicin Skåne, Clinical Chemistry in Lund, Lund, Sweden; 8 Departments of Laboratory Medicine, Medical Epidemiology & Biostatistics, Karolinska Institutet and Karolinska Hospital, Stockholm, Sweden

## Abstract

**Background:**

Breast cancer today has many established risk factors, both genetic and environmental, but these risk factors by themselves explain only part of the total cancer incidence. We have investigated potential interactions between certain known genetic and phenotypic risk factors, specifically nine single nucleotide polymorphisms (SNPs) and height, body mass index (BMI) and hormone replacement therapy (HRT).

**Methods:**

We analyzed samples from three different study populations: two prospectively followed Swedish cohorts and one Icelandic case–control study. Totally 2884 invasive breast cancer cases and 4508 controls were analysed in the study. Genotypes were determined using Mass spectrometry-Maldi-TOF and phenotypic variables were derived from measurements and/or questionnaires. Odds Ratios and 95% confidence intervals were calculated using unconditional logistic regression with the inclusion of an interaction term in the logistic regression model.

**Results:**

One SNP (rs851987 in ESR1) tended to interact with height, with an increasingly protective effect of the major allele in taller women (p = 0.007) and rs13281615 (on 8q24) tended to confer risk only in non users of HRT (p-for interaction = 0.03). There were no significant interactions after correction for multiple testing.

**Conclusions:**

We conclude that much larger sample sets would be necessary to demonstrate interactions between low-risk genetic polymorphisms and the phenotypic variables height, BMI and HRT on the risk for breast cancer. However the present hypothesis-generating study has identified tendencies that would be of interest to evaluate for gene-environment interactions in independent materials.

## Background

Genome wide association studies (GWAS), have discovered several new genetic polymorphisms affecting breast cancer risk [1-3]. Even though these individual risk-factors each confer quite small increases in risk, a positive association is seen between the number of risk alleles carried and risk for breast cancer [4,5].

The phenotypic variables height, body mass index (BMI) and use of hormone replacement therapy (HRT) reflect to varying degrees genetic background and environmental exposure. Both height and BMI have previously been shown to associate with breast cancer [6,7]. Increase in height has been shown to yield a proportional increase in breast cancer risk and obese women have a greater risk to contract postmenopausal breast cancer. Increased risk is also established for users of HRT [7], which has been speculated to interact with low-risk polymorphisms in the FGFR2 gene [8,9].

Although there have been investigations on gene-environment interactions in breast cancer [10], this area remains to a large extent unexplored.

The aim of this study was to investigate if height, BMI and HRT modify the genetic predisposition to breast cancer conferred by reported low-risk polymorphisms. For this purpose we had access to two well defined Swedish population based cohorts as well as an Icelandic hospital based case control study, altogether 7738 samples (3016 cases and 4722 controls).

## Methods

### Study populations

The samples originate from two Swedish independent population based cohorts; the Malmö Diet and Cancer Study (MDCS) from southern Sweden and the North Sweden Health and Disease Study (NSHDS), together comprising 2410 incident cases and 3829 controls. The third sample collection was an Icelandic population-based case control study including 866 cases and 948 controls. Written informed consent was retrieved from all women prior to donating their samples. All cohorts have been described previously [11] and are briefly presented below.

#### MDCS

The Malmö Diet and Cancer Study (MDCS) is a prospective cohort study initiated in 1991. Totally it comprises 17035 female residents of Malmö Sweden recruited between 1991 and 1996 [12,13]. By linkage to the national cancer registry until 31^st^ of December 2007, 730 incident cases of invasive breast cancer were identified among MDCS participants. They were matched to 1460 controls from the same cohort according to sex, age (+/− 6 months), and date of sampling at baseline (+/− 2 months). Median age at breast cancer diagnosis was 65 years (range 45–84). Thirty-three cases and 65 controls were ≤50 years of age at time of diagnosis.

The MDCS and the present analyses were approved by the Ethical Committee at Lund University (LU 51–90, Dnr 652/2005 and Dnr 2009/682).

#### NSHDS

The Northern Sweden Health and Disease Study (NSHDS) include the Västerbotten Intervention Program (VIP), and the Mammography Screening Program (MSP), initiated in 1985 and 1995 respectively. Participants in the VIP are screened at 40, 50 and 60 years of age and mammography screening and blood sampling is performed among women between 50 and 69 years of age [14]. Through linkage with the cancer register up to December 31^st^, 2008, 1680 prospective cases of invasive breast cancer (median age 56 years, range 27–95) were identified. They were matched to 2314 controls by sex, age (+/− 6 months), and date of sampling at baseline (+/− 2 months), (474 cases and 606 controls ≤50 years of age). Information on HRT use was available for 1420 of these cases.

The NSHDS and the present analyses were approved by the Ethical Committee at Umeå University (Dnr: 2010-147-132 and 07–141).

#### ICELAND

The Icelandic samples were collected between 1998 and 2006 and represent 45–77% of all Icelandic women with invasive breast cancer diagnosed between 1957 and 2007. The rate of participation varied somewhat depending on the year of diagnosis and was highest between 1999 and 2003 (77%). Unmatched controls were collected between 2000 and 2004, either from women who participated in the population-based cervical or breast cancer screening program and found free of breast cancer or from older women in retirement homes who had not been diagnosed with breast cancer, to generally reflect the ages of the cases. By linkage to the Icelandic cancer registry in 2008 we identified cases diagnosed before 31^st^ of December 2007. Totally 866 cases (median age 55 years, range 22–98, 314 ≤ 50 years) and 948 controls (median age 58 years, range 25–102, 256 ≤ 50 years) had DNA available and were eligible to us.

The use of these samples was approved by the data protection law (200605037), and the Icelandic Science Ethics Committee (VSNb2006050001/03-16 and VSNb2005070008/03-16).

### Data collection

Participants in both Swedish cohorts completed a questionnaire providing information about current medication at the time of recruitment. Participants in the NSHDS also provided information about height and weight while a trained nurse at the study centre measured height and weight, for participants in MDCS [15].

The Icelandic women answered questions about height, weight and HRT use when they attended the Detection Cancer Clinic (breast cancer mammography or cervical screening) at the Icelandic Cancer Society. The women answered questions at least every tenth year and the most recent answers were used in the study. For the Icelandic cases only data collected prior to breast cancer diagnosis was used. BMI for all participants was calculated as kg/m^2^.

### SNP selection

**Figure 1:**
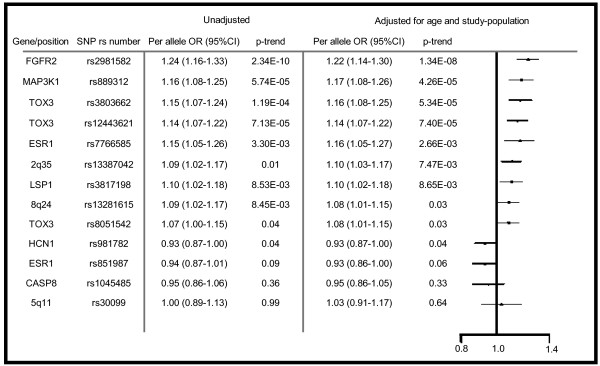
**Odds Ratios and Confidence Intervals for all SNPs.** All 13 primary polymorphisms and their respective OR and p-value in this sample set. Squares represent OR and brackets represent 95% CI for samples adjusted for age and study population. A subset of previously published data [11].

All loci identified by GWAS to be associated with breast cancer and published before June 31^st^ 2007 were initially included in the study [1-3]. Individual SNPs were selected from the publications by Easton et al. and Stacey et al. This primary selection included 10 SNPs, as well as one SNP in CASP8 identified using the candidate gene approach [16]. Two SNPs selected from our own candidate CpG SNP study [11] were also included making a total of 13 SNPs (Figure 1).

### Assay design and genotyping

Eleven SNPs, combined by the SEQUENOM MassARRAY® Designer software in a single multiplex assay were analyzed on a MALDI-TOF mass spectrometer (SEQUENOM MassArray) using standard iPLEX reagents and protocol (SEQUENOM) and 10 ng DNA as PCR template. Primer sets were from Metabion (Martinsried, Germany).

SNPs rs2981582 and rs1045485 were analyzed by a separate TaqMan® “assay by design” genotyping assay on a 7900HT instrument, using Master mix No UNG from Applied Biosystems according to the manufacturer’s instructions. Reaction mixtures (6μL) containing 2 ng of DNA template, primers (rs2981582 forward primer 5′-CAG CAC TCA TCG CCA CTT AAT G-3′, reverse primer 5′-GAC ACC ACT CGG ACT GCT-3′, and probes 5′-VIC-TCT CCG CAA ACA GG-MGB-3′ and 5′-FAM-CTC TCC ACA AAC AGG-MGB-3′) (rs1045485 forward primer 5′-ACC ACG ACC TTT GAA GAG CTT -3′, reverse primer 5′-ACT GTG GTC CAT GAG TTG GTA GAT-3′, and probes 5′-VIC-CCC CAC GAT GAC TG-MGB-3′ and 5′-FAM-CCC CAC CAT GAC TG-MGB-3′) were subjected to two minutes at 50°C and ten minutes at 95°C, followed by 50 PCR cycles of 95°C for 15 seconds and 60°C for one minute.

Three percent of the samples from NSHDS and five percent of the Icelandic samples were included as blinded duplicates for quality control purposes.

### Statistical analysis

Individual samples producing results in < 80% of the assays were excluded prior to statistical analyses to eliminate samples with low-quality DNA. Genotype data from control samples were tested for consistency with Hardy-Weinberg equilibrium (HWE) using a χ² p-value cutpoint of 0.001. Unconditional logistic regression was used to measure the independent association between each genotype and breast cancer, with Odds ratios and 95% confidence intervals (CI) estimated for each genotype. Per allele OR (p-trend) was calculated using 0, 1 or 2 copies of the minor allele (a) as a continuous variable. OR and 95% CI were calculated between each phenotypic variable (Height, BMI and HRT) and risk for breast cancer, these results were also age adjusted. Data was then stratified into tertiles according to height (<162 cm, 162–166 am and >166 cm), and into subcategories of BMI according to the WHO guidelines (Normal weight: 18.5-25, Overweight: 25–30 and Obese > 30). For HRT subjects data was stratified according to reported “non use” and “current use”. The current users were further divided into users of only estrogen or combined hormones. OR and 95%CI were calculated for each variable (Height, BMI and HRT) and risk for breast cancer.

A p-value for interaction was estimated for each pair of genotype/phenotype and a value of less than 0.05 was considered statistically significant. As adjustment for multiple comparisons this value was divided by the number of interaction analyses, according to Bonferroni, (8 SNPs x 3 =24) and the new significance threshold was 0.002. All results were adjusted for age and study population.

## Results

Of the initial 7738 samples selected for the project 7392 (95.5%) were successfully retrieved and genotyped for ≥ 80% of the SNPs. All SNPs had a genotyping success rate > 94%, with an average of 98.0%. Results of all 200 analyses performed on duplicate samples were in 100% concordance.

Per allele OR for each independent SNP is presented in Figure 1. Ten of the SNPs were significant (p < 0.05) in our material with rs2981852 (FGFR2), rs889312 (MAP3K1) and rs3803662 (TOX3) exhibiting the highest ORs. Two of the SNPs had p-values >0.1 (rs1045485 [CASP8] and rs30099 [5q11]) and were excluded from further analysis.

Three of the SNPs in TOX3 (rs3803662, rs12443621 and rs8051542) exhibited linkage (results not shown) as has previously been reported [1,4]. Rs12443621 and rs80515442 were therefore excluded from further analysis.

Independent analysis of risk association with each phenotypic variable (height, BMI, HRT) within the entire study population revealed a significantly increased risk of breast cancer for individuals >162 cm compared to shorter women, this association was weakened following age adjustment. No statistical significant correlation between BMI and risk for breast cancer was found in this population. For current use vs. non-use of HRT, a significantly increased risk was seen for users, OR (95% CI) 1.24 (1.08-1.42), which remained after adjustment for age (Table 1).

**Table 1:** Environmental risk factors (MDCS, NSHDS and ICELAND)

**Risk factor**	**Categories**	**MDCS**		**NSHDS**		**ICELAND**		**TOTAL**	
		**Count**	OR*	**Count**	OR*	**Count**	OR*	**Count**	OR*
		**Case/Control**	**(95% CI)**	**Case/Control**	**(95% CI)**	**Case/Control**	**(95% CI)**	**Case/Control**	**(95% CI)**
**All individuals**									
BMI	Normal Weight	334/695	1	637/997	1	361/326	1	1332/2018	1
	Over weight	237/450	1.26 (1.02-1.56)	439/701	0.99 (0.84-1.15)	241/312	0.76 (0.60-0.96)	917/1463	1.00 (0.90-1.12)
	Obese	95/194	1.25 (0.94-1.66)	167/286	0.91 (0.74-1.14)	112/139	0.79 (0.59-1.07)	374/619	0.97 (0.84-1.13)
Height	<162 cm	207/471	1	398/722	1	171/173	1	776/1366	1
	162-166 cm	233/443	1.03 (0.81-1.30)	445/657	1.21 (1.02-1.44)	289/307	0.92 (0.70-1.20)	967/1407	1.15 (1.02-1.30)
	>166 cm	233/448	0.93 (0.73-1.18)	436/640	1.21 (1.01-1.44)	354/414	0.78 (0.60-1.02)	1023/1502	1.09 (0.97-1.23)
**> 50 years**									
HRT	Non users	341/806	1	777/1320	1	66/286	1	1184/2412	1
	Current users	181/256	1.26 (0.99-1.61)	186/295	1.09 (0.89-1.34)	105/225	2.06 (1.45-2.95)	424/776	1.22 (1.07-1.39)
BMI	Normal Weight	322/694	1	400/682	1	189/217	1	911/1593	1
	Over weight	232/449	1.26 (1.02-1.57)	320/504	1.09 (0.91-1.32)	188/255	0.85 (0.65-1.12)	740/1208	1.12 (0.99-1.26)
	Obese	95/194	1.26 (0.94-1.68)	130/205	1.10 (0.85-1.41)	86/109	0.91 (0.65-1.29)	311/508	1.13 (0.96-1.34)
Height	<162 cm	205/471	1	283/534	1	125/136	1	613/1141	1
	162-166 cm	224/442	1.02 (0.81-1.29)	311/458	1.26 (1.03-1.55)	198/244	0.88 (0.64-1.19)	733/1144	1.13 (0.99-1.30)
	>166 cm	227/447	0.94 (0.74-1.19)	279/421	1.22 (0.99-1.51)	199/274	0.78 (0.57-1.06)	705/1142	1.05 (0.92-1.21)

*Adjusted for age.

### Stratified analysis and interactions

After stratification by height (as described in materials and methods), one SNP (rs851987) in ESR1 had a p-interaction = 0.007 with height, with an increasingly protective effect of the major allele in taller women, but it did not pass the threshold for multiple comparisons (p = 0.002) (Table 2).

**Table 2:** **OR adjusted for Age and Study Population, stratified byHeight**

**SNP rs nr**	**Genotype**	**p-value for interaction**	**All individuals**		**Height <162.0 cm**		**Height 162.0-166.0 cm**		**Height >166.0 cm**	
			**case/contr: 2869/4480**		**case/control: 776/1366**		**case/control: 967/1407**		**case/control: 1023/1502**	
			**OR 95%CI**	**p-trend**	**OR 95%CI**	**p-trend**	**OR 95%CI**	**p-trend**	**OR 95%CI**	**p-trend**
FGFR2 2981582	AA		**1**		1		1		1	
	Aa		**1.23 (1.10-1.37)**		1.35 (1.10-1.66)		1.33 (1.10-1.61)		1.07 (0.89-1.28)	
	aa		**1.48 (1.29-1.69)**		1.57 (1.21-2.02)		1.56 (1.22-1.99)		1.32 (1.04-1.67)	
	Per Allele	0,41	**1.22 (1.14-1.30)**	**1.34E-08**	1.26 (1.11-1.43)	2.86E-04	1.26 (1.12-1.42)	1.65E-04	1.14 (1.01-1.28)	0.03
TNR09 3803662	AA		**1**		1		1		1	
	Aa		**1.14 (1.03-1.26)**		1.09 (0.90-1.32)		1.23 (1.03-1.47)		1.15 (0.97-1.36)	
	aa		**1.39 (1.17-1.67)**		1.37 (1.00-1.87)		1.37 (1.00-1.89)		1.42 (1.03-1.95)	
	Per Allele	0,91	**1.16 (1.08-1.25)**	**5.34E-05**	1.14 (1.00-1.31)	0.06	1.20 (1.05-1.37)	6.78E-03	1.17 (1.03-1.33)	0.02
MAP3K1 889312	AA		**1**		1		1		1	
	Aa		**1.21 (1.09-1.34)**		1.29 (1.06-1.55)		1.14 (0.95-1.36)		1.18 (1.00-1.40)	
	aa		**1.29 (1.09-1.54)**		1.31 (0.95-1.81)		1.05 (0.76-1.45)		1.53 (1.14-2.06)	
	Per Allele	0,45	**1.17 (1.08-1.26)**	**4.26E-05**	1.20 (1.04-1.37)	9.92E-03	1.07 (0.94-1.22)	0.29	1.22 (1.07-1.38)	2.20E-03
8q24 13281615	AA		**1**		1		1		1	
	Aa		**1.12 (1.00-1.24)**		1.21 (0.98-1.48)		1.07 (0.89-1.29)		1.12 (0.93-1.34)	
	aa		**1.15 (1.00-1.32)**		1.24 (0.96-1.60)		1.17 (0.92-1.49)		1.09 (0.87-1.38)	
	Per Allele	0,90	**1.08 (1.01-1.15)**	**0.03**	1.12 (0.99-1.27)	0.07	1.08 (0.96-1.22)	0.20	1.06 (0.94-1.18)	0.35
LSP1 3817198	AA		**1**		1		1		1	
	Aa		**1.12 (1.02-1.24)**		1.19 (0.99-1.45)		1.08 (0.90-1.28)		1.08 (0.91-1.28)	
	aa		**1.19 (1.00-1.40)**		1.50 (1.11-2.02)		1.14 (0.84-1.55)		1.04 (0.79-1.38)	
	Per Allele	0,48	**1.10 (1.02-1.18)**	**8.65E-03**	1.21 (1.06-1.39)	4.41E-03	1.07 (0.94-1.22)	0.29	1.04 (0.92-1.18)	0.50
2q35	AA		**1**		1		1		1	
	Aa		**1.05 (0.93-1.19)**		0.90 (0.72-1.14)		1.21 (0.98-1.50)		0.97 (0.79-1.19)	
	aa		**1.20 (1.05-1.37)**		1.09 (0.85-1.40)		1.28 (1.01-1.63)		1.21 (0.96-1.52)	
	Per Allele	0,38	**1.10 (1.03-1.17)**	**7.47E-03**	1.05 (0.93-1.19)	0.43	1.13 (1.00-1.27)	0.04	1.10 (0.98-1.23)	0.11
ESR1 7766585	AA		**1**		1		1		1	
	Aa		**1.23 (1.10-1.37)**		1.12 (0.90-1.38)		1.26 (1.03-1.53)		1.25 (1.04-1.50)	
	aa		**1.02 (0.74-1.41)**		0.83 (0.43-1.58)		1.21 (0.69-2.13)		0.98 (0.57-1.66)	
	Per Allele	0,83	**1.16 (1.05-1.27)**	**2.66E-03**	1.05 (0.88-1.26)	0.60	1.20 (1.02-1.42)	0.03	1.16 (0.99-1.36)	0.06
HCN1 981782	AA		**1**		1		1		1	
	Aa		**0.97 (0.87-1.09)**		0.92 (0.74-1.14)		1.03 (0.84-1.26)		1.00 (0.82-1.21)	
	aa		**0.86 (0.75-0.99)**		0.91 (0.70-1.18)		0.96 (0.75-1.22)		0.77 (0.61-0.98)	
	Per Allele	0,51	**0.93 (0.87-1.00)**	**0.04**	0.95 (0.83-1.08)	0.44	0.98 (0.87-1.11)	0.73	0.88 (0.78-0.99)	0.04
ESR1 851987	AA		**1**		1		1		1	
	Aa		**0.91 (0.82-1.02)**		0.96 (0.78-1.17)		0.92 (0.76-1.11)		0.87 (0.73-1.05)	
	aa		**0.88 (0.74-1.05)**		1.44 (1.06-1.95)		0.69 (0.50-0.96)		0.71 (0.52-0.98)	
	Per Allele	**6.70E-03**	**0.93 (0.86-1.00)**	**0.06**	1.12 (0.97-1.28)	0.12	0.86 (0.75-0.99)	0.04	0.86 (0.75-0.98)	0.02

None of the SNPs showed any tendencies towards significant interactions after stratification according to BMI (Table 3).

None of the SNPs showed any tendencies towards significant interactions after stratification according to BMI (Table 3).

**Table 3:** **OR adjusted for Age and Study Population, stratified byBMI**

**SNP rs nr**	**Genotype**	**p-value for interaction**	**All individuals**		**Normal Weigth**		**Over Weight**		**Obese**	
					**BMI = 18.5-24.99**		**BMI = 25.00-29.99**		**BMI ≥ 30**	
			**case/contr: 2869/4480**		**case/control: 1332/2018**		**case/control: 917/1463**		**case/control: 374/619**	
			**OR 95%CI**	**p-trend**	**OR 95%CI**	**p-trend**	**OR 95%CI**	**p-trend**	**OR 95%CI**	**p-trend**
FGFR2 2981582	AA		**1**		1		1		1	
	Aa		**1.23 (1.10-1.37)**		1.26 (1.07-1.48)		1.18 (0.97-1.42)		1.08 (0.80-1.46)	
	aa		**1.48 (1.29-1.69)**		1.46 (1.18-1.80)		1.57 (1.24-1.99)		1.14 (0.78-1.66)	
	Per Allele	0.59	**1.22 (1.14-1.30)**	**1.34E-08**	1.21 (1.10-1.35)	2.03E-04	1.25 (1.11-1.40)	2.20E-04	1.07 (0.89-1.29)	0.49
TNR09 3803662	AA		**1**		1		1		1	
	Aa		**1.14 (1.03-1.26)**		1.16 (1.00-1.35)		1.13 (0.94-1.34)		1.27 (0.97-1.67)	
	aa		**1.39 (1.17-1.67)**		1.44 (1.10-1.89)		1.21 (0.89-1.65)		1.72 (1.07-2.78)	
	Per Allele	0.72	**1.16 (1.08-1.25)**	**5.34E-05**	1.18 (1.06-1.32)	2.88E-03	1.11 (0.98-1.26)	0.11	1.30 (1.06-1.58)	0.01
MAP3K1 889312	AA		**1**		1		1		1	
	Aa		**1.21 (1.09-1.34)**		1.15 (0.99-1.33)		1.33 (1.11-1.58)		1.21 (0.92-1.59)	
	aa		**1.29 (1.09-1.54)**		1.22 (0.94-1.59)		1.63 (1.20-2.22)		1.22 (0.76-1.97)	
	Per Allele	0.47	**1.17 (1.08-1.26)**	**4.26E-05**	1.12 (1.01-1.25)	0.04	1.30 (1.14-1.48)	8.64E-05	1.15 (0.94-1.40)	0.18
8q24 13281615	AA		**1**		1		1		1	
	Aa		**1.12 (1.00-1.24)**		1.18 (1.01-1.39)		1.04 (0.86-1.25)		1.13 (0.84-1.52)	
	aa		**1.15 (1.00-1.32)**		1.22 (0.99-1.49)		1.14 (0.90-1.45)		1.10 (0.75-1.60)	
	Per Allele	0.82	**1.08 (1.01-1.15)**	**0.03**	1.11 (1.01-1.23)	0.04	1.06 (0.95-1.20)	0.30	1.06 (0.88-1.27)	0.54
LSP1 3817198	AA		**1**		1		1		1	
	Aa		**1.12 (1.02-1.24)**		1.16 (0.99-1.34)		1.11 (0.93-1.33)		0.94 (0.72-1.24)	
	aa		**1.19 (1.00-1.40)**		1.41 (1.10-1.81)		1.06 (0.80-1.42)		0.83 (0.50-1.38)	
	Per Allele	0.35	**1.10 (1.02-1.18)**	**8.65E-03**	1.18 (1.06-1.31)	3.22E-03	1.06 (0.93-1.20)	0.38	0.93 (0.75-1.14)	0.47
2q35	AA		**1**		1		1		1	
	Aa		**1.05 (0.93-1.19)**		1.04 (0.87-1.24)		1.00 (0.81-1.23)		1.12 (0.80-1.58)	
	aa		**1.20 (1.05-1.37)**		1.09 (0.89-1.33)		1.28 (1.01-1.61)		1.38 (0.94-2.03)	
	Per Allele	0.63	**1.10 (1.03-1.17)**	**7.47E-03**	1.04 (0.94-1.15)	0.40	1.13 (1.01-1.27)	0.04	1.18 (0.97-1.43)	0.09
ESR1 7766585	AA		**1**		1		1		1	
	Aa		**1.23 (1.10-1.37)**		1.29 (1.09-1.52)		1.18 (0.97-1.43)		1.07 (0.79-1.44)	
	aa		**1.02 (0.74-1.41)**		0.98 (0.60-1.61)		1.27 (0.76-2.14)		0.51 (0.18-1.41)	
	Per Allele	0.47	**1.16 (1.05-1.27)**	**2.66E-03**	1.19 (1.03-1.37)	0.02	1.16 (0.99-1.37)	0.07	0.96 (0.74-1.24)	0.75
HCN1 981782	AA		**1**		1		1		1	
	Aa		**0.97 (0.87-1.09)**		1.04 (0.87-1.24)		1.01 (0.82-1.23)		0.87 (0.63-1.20)	
	aa		**0.86 (0.75-0.99)**		0.84 (0.68-1.04)		1.00 (0.79-1.28)		0.87 (0.59-1.27)	
	Per Allele	0.50	**0.93 (0.87-1.00)**	**0.04**	0.92 (0.83-1.02)	0.13	1.00 (0.89-1.13)	0.98	0.93 (0.77-1.13)	0.46
ESR1 851987	AA		**1**		1		1		1	
	Aa		**0.91 (0.82-1.02)**		0.91 (0.78-1.06)		0.97 (0.80-1.17)		0.92 (0.69-1.22)	
	aa		**0.88 (0.74-1.05)**		0.92 (0.71-1.20)		0.83 (0.61-1.13)		0.76 (0.46-1.25)	
	Per Allele	0.89	**0.93 (0.86-1.00)**	**0.06**	0.94 (0.84-1.05)	0.29	0.93 (0.81-1.07)	0.30	0.89 (0.72-1.10)	0.28

Following stratification of genotypes according to reported current use or non-use of hormone replacement therapy, rs13281615 (8q24) was significant only in non users of HRT with a p-for interaction of 0.03, indicating borderline significance (Table 4).

**Table 4:** **OR adjusted for Age and Study Population, stratified byHRT**

**SNP rs nr**	**Genotype**	**p-value for interaction**	All individuals*		HRT Non Users*		HRT Current Users*		HRT Estrogen*		HRT Comb*	
			**case/contr: 2869/4480**		**case/contr: 1536/2949**		**case/contr: 494/800**		**case/contr: 185/324**		**case/contr:298/455**	
			**OR 95%CI**	**p-trend**	**OR 95%CI**	**p-trend**	**OR 95%CI**	**p-trend**	**OR 95%CI**	**p-trend**	**OR 95%CI**	**p-trend**
FGFR2 2981582	AA		**1**		1		1		1		1	
	Aa		**1.23 (1.10-1.37)**		1.22 (1.06-1.41)		1.16 (0.89-1.50)		1.15 (0.75-1.75)		1.17 (0.83-1.65)	
	aa		**1.48 (1.29-1.69)**		1.55 (1.29-1.89)		1.63 (1.17-2.26)		1.44 (0.86-2.42)		1.87 (1.21-2.89)	
	Per Allele	0,83	**1.22 (1.14-1.30)**	**1.34E-08**	1.24 (1.14-1.36)	2.04E-06	1.26 (1.07-1.49)	4.83E-03	1.20 (0.92-1.55)	0.18	1.34 (1.08-1.66)	7.30E-03
TNRC9 3803662	AA		**1**		1		1		1		1	
	Aa		**1.14 (1.03-1.26)**		1.15 (1.01-1.31)		1.17 (0.91-1.49)		1.31 (0.89-1.94)		1.13 (0.83-1.56)	
	aa		**1.39 (1.17-1.67)**		1.48 (1.18-1.86)		1.47 (0.96-2.27)		1.56 (0.79-3.11)		1.53 (0.86-2.69)	
	Per Allele	0,98	**1.16 (1.08-1.25)**	**5.34E-05**	1.19 (1.08-1.31)	3.74E-04	1.19 (1.00-1.43)	0.05	1.28 (0.96-1.70)	0.09	1.19 (0.94-1.51)	0.15
MAP3K1 889312	AA		**1**		1		1		1		1	
	Aa		**1.21 (1.09-1.34)**		1.27 (1.11-1.45)		1.04 (0.82-1.32)		0.87 (0.59-1.29)		1.13 (0.82-1.54)	
	aa		**1.29 (1.09-1.54)**		1.20 (0.95-1.53)		1.54 (1.02-2.33)		1.81 (0.98-3.33)		1.31 (0.74-2.33)	
	Per Allele	0,12	**1.17 (1.08-1.26)**	**4.26E-05**	1.17 (1.06-1.29)	1.90E-03	1.15 (0.97-1.38)	0.11	1.16 (0.88-1.52)	0.28	1.14 (0.90-1.44)	0.29
8q24 13281615	AA		**1**		1		1		1		1	
	Aa		**1.12 (1.00-1.24)**		1.20 (1.04-1.39)		1.05 (0.81-1.34)		0.90 (0.60-1.36)		1.10 (0.80-1.53)	
	aa		**1.15 (1.00-1.32)**		1.43 (1.20-1.71)		0.86 (0.61-1.20)		0.88 (0.52-1.51)		0.84 (0.53-1.32)	
	Per Allele	**0,03**	**1.08 (1.01-1.15)**	**0.03**	1.20 (1.10-1.31)	6.10E-05	0.95 (0.81-1.12)	0.52	0.93 (0.72-1.22)	0.61	0.95 (0.77-1.18)	0.67
LSP1 3817198	AA		**1**		1		1		1		1	
	Aa		**1.12 (1.02-1.24)**		1.13 (0.99-1.29)		1.29 (1.01-1.65)		1.32 (0.89-1.94)		1.30 (0.95-1.80)	
	aa		**1.19 (1.00-1.40)**		1.06 (0.85-1.31)		1.68 (1.12-2.53)		1.19 (0.61-2.33)		2.35 (1.37-4.03)	
	Per Allele	0,13	**1.10 (1.02-1.18)**	**8.65E-03**	1.06 (0.97-1.17)	0.21	1.30 (1.09-1.55)	4.07E-03	1.18 (0.89-1.56)	0.26	1.44 (1.14-1.82)	2.16E-03
2q35	AA		**1**		1		1		1		1	
	Aa		**1.05 (0.93-1.19)**		1.05 (0.89-1.23)		0.95 (0.72-1.27)		0.77 (0.48-1.25)		1.13 (0.78-1.63)	
	aa		**1.20 (1.05-1.37)**		1.15 (0.97-1.38)		1.20 (0.87-1.67)		1.26 (0.75-2.10)		1.20 (0.78-1.85)	
	Per Allele	0,68	**1.10 (1.03-1.17)**	**7.47E-03**	1.08 (0.98-1.17)	0.11	1.10 (0.93-1.29)	0.26	1.14 (0.88-1.48)	0.33	1.10 (0.88-1.36)	0.40
ESR1 7766585	AA		**1**		1		1		1		1	
	Aa		**1.23 (1.10-1.37)**		1.23 (1.07-1.43)		1.05 (0.81-1.36)		1.31 (0.85-2.03)		0.88 (0.63-1.23)	
	aa		**1.02 (0.74-1.41)**		1.01 (0.67-1.53)		1.29 (0.61-2.73)		1.43 (0.52-3.93)		0.86 (0.26-2.81)	
	Per Allele	0,47	**1.16 (1.05-1.27)**	**2.66E-03**	1.15 (1.02-1.31)	0.02	1.07 (0.86-1.34)	0.53	1.26 (0.89-1.79)	0.19	0.89 (0.66-1.21)	0.46
HCN1 981782	AA		**1**		1		1		1		1	
	Aa		**0.97 (0.87-1.09)**		0.88 (0.76-1.02)		1.15 (0.86-1.53)		0.88 (0.57-1.38)		1.34 (0.92-1.96)	
	aa		**0.86 (0.75-0.99)**		0.80 (0.66-0.95)		0.93 (0.66-1.31)		0.74 (0.43-1.28)		1.05 (0.67-1.64)	
	Per Allele	0,26	**0.93 (0.87-1.00)**	**0.04**	0.89 (0.81-0.98)	0.01	0.97 (0.82-1.14)	0.70	0.86 (0.66-1.13)	0.28	1.02 (0.82-1.27)	0.84
ESR1 851987	AA		**1**		1		1		1		1	
	Aa		**0.91 (0.82-1.02)**		0.94 (0.82-1.07)		0.80 (0.62-1.04)		0.94 (0.63-1.43)		0.73 (0.52-1.03)	
	aa		**0.88 (0.74-1.05)**		0.90 (0.72-1.13)		0.93 (0.62-1.42)		0.88 (0.46-1.68)		0.96 (0.54-1.69)	
	Per Allele	0,52	**0.93 (0.86-1.00)**	**0.06**	0.94 (0.86-1.04)	0.24	0.90 (0.75-1.09)	0.28	0.94 (0.70-1.25)	0.67	0.87 (0.68-1.12)	0.29

## Discussion

In this study we have explored interactions between reported genetic risk factors for breast cancer and the three additional established risk factors; height, BMI and HRT in 2884 cases and 4508 controls. The strongest tendency for interaction found was that between height and rs851987 in ESR1, although it did not pass the threshold for multiple comparisons. Taller women carrying the T-allele appeared to have reduced breast cancer risk (p for interaction = 0.007) (Table 2). Rs851987 was described by Harlid et al. [11] and is situated in the far end of the extended promoter region of ESR1, about 3.7 kb 5′ of exon F. Exon F and its promoter were originally described by Thompson et al. [17] and have later been shown to affect the level of ESR1 expression in osteoblastic cells [18,19]. A potential association between ESR1 and height has been described in another study comprising adult males from two Swedish population cohorts [20]. Mutations in ESR1 have been reported to delay fusion of the epiphyseal plates at puberty [21], and one may speculate that rs851987 either participates in this biological effect or is linked to other causal variants.

One SNP (rs13281615 in 8q4) first described by Easton et al. [1] showed a weak tendency for interaction with use of HRT. The minor allele seems to confer increased breast cancer risk in HRT non-users but no excess risk in current users. The association in non-users is strong with a per-allele OR (95%CI) of 1.20 (1.10-1.31) (p-trend = 6.1 × 10^-5^) compared to a per-allele OR (95%CI) of 1.08 (1.01-1.15) (p-trend = 0.03) in all users. The SNP is situated in region 8q24 that contains no known genes but is in close proximity to FAM84B (coding for a breast cancer membrane associated protein) and the proto-oncogene MYC. The 8q24 locus has previously been reported to associate with other types of cancer in addition to breast cancer [22] and to be more strongly associated with ER + than ER- tumours [23].

Since the first GWAS on breast cancer was published in 2007 several replication and interaction studies of varying sizes have been published [24-27]. In 2010, a large interaction study comprising 7610 breast cancer cases from the Million Women Study in UK was undertaken and potential interactions between 12 different SNPs and 10 different variables (including height, BMI and HRT) were tested [10]. This study did not find (contrary to previous suggestions) any significant gene-environment interactions. Our study originally included ten of the same polymorphisms as in the Million Women Study (excluding rs1982073 in TGFB1 and rs1800054 in ATM), but also included one additional SNP from Easton et al. [1] and two additional SNPs from our own candidate CpG study [11] (rs7766585 and rs851987 both in ESR1). Although our material is not as large, our study is comprised of three well described study-populations, two of which were prospectively followed for breast cancer incidence using the comprehensive, population-based Swedish Cancer Registry [28]. Thus, our complete case ascertainment and ability to select matched controls from the same study base is likely to have resulted in low risk for selection biases. However, the intervals between data collection, blood sampling, and diagnosis differ substantially between the three different study populations, something that might be considered a limitation of the study.

Considering demographic traits, participants in the MDCS have a slightly higher socioeconomic status than the general population, but as this selection is the same for the study base from which cases and controls are derived, it should not affect the validity of our study [13]. MDCS participants were recruited at age 45–65 years. The exclusion of prevalent cases removes early breast cancer cases from this population. While the NHSDS participants were primarily included from age 40 and upwards, mammography screening had identified some cases as young as 27 years. In Iceland prevalent cases of breast cancer were recruited at varying times after diagnosis, resulting in an exclusion of early lethal cases and older women with other causes of death. As the Icelandic controls were collected later and from the same sample population as the cases there is the possibility of selection bias. Another limitation of our study is the fact that HRT is reported only once (at recruitment) without information about duration. We also lacked information about other risk factors than age, height, BMI, HRT and therefore could not adjust our results for other potential confounders.

## Conclusions

Our evaluation of genetic predisposition for breast cancer in relation to three different environmental risk factors found no significant gene-environment interactions. We did find tendencies for certain SNPs to exert an effect on breast cancer risk only in women with certain phenotypes. In particular the potential interaction between height and rs851987 in ESR1 in relation to breast cancer risk could merit further investigation. However, independent studies with many more cases would be needed to verify this finding.

## Competing interests

The authors declare that they have no competing interests.

## Authors’ contributions

SH had full access to all the data and in the study and takes responsibility for the integrity of the data and the accuracy of the data analysis. SH and JC are the principal investigators of the study and were responsible for the planning of the study. JD participated in the conception and design of the study and contributed with supervising, funding, and administrative support. MILI participated in designing the study. JEE and PL contributed samples to the study. SH and MILI performed all genetic analysis. SH analyzed the data (data extraction and statistical analysis). SH, JC, JD, SB and JM interpreted the data. SH performed the literature search. SH wrote; JC, JD, JM revised; MILI, SB, JEE, and PL reviewed the paper and contributed suggestions for improvement that were implemented. All authors approved the final version.

## Pre-publication history

The pre-publication history for this paper can be accessed here:

http://www.biomedcentral.com/1472-6874/12/17/prepub
